# (*E*)-*N*′-[(2-Hy­droxy­naphthalen-1-yl)methyl­idene]-4-methyl­benzene­sulfono­hydrazide

**DOI:** 10.1107/S1600536811006088

**Published:** 2011-02-26

**Authors:** Gholam Hossein Shahverdizadeh, Rahman Bikas, Maryam Eivazi, Parisa Mahboubi Anarjan, Behrouz Notash

**Affiliations:** a Department of Applied Chemistry, Research Laboratory, Islamic Azad University, Tabriz Branch, Tabriz, Iran; b Department of Chemistry, Shahid Beheshti University, G. C., Evin, Tehran 1983963113, Iran

## Abstract

In the title compound, C_18_H_16_N_2_O_3_S, the dihedral angle between the planes of the benzene ring and the naphthyl ring system is 83.37 (10)°. An intra­molecular O—H⋯N hydrogen bond occurs. Inter­molecular N—H⋯O hydrogen bonds stabilize the crystal structure. There is a π–π inter­action between the naphthyl ring systems [centroid–centroid distance = 3.7556 (15) Å]. In addition, naphth­yl–tolyl and naphth­yl–naphthyl C—H⋯π inter­actions are observed.

## Related literature

For related structures, see: Bikas *et al.* (2010 ▶); Silva *et al.* (2006 ▶); Zimmer *et al.* (1959 ▶).
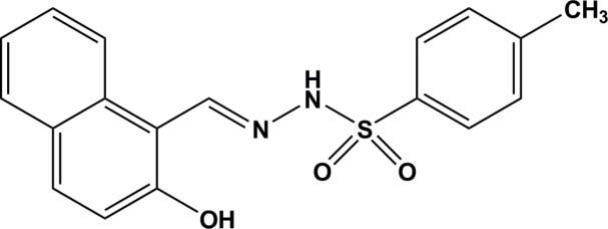


## Experimental

### 

#### Crystal data

C_18_H_16_N_2_O_3_S
*M* *_r_* = 340.40
Monoclinic, 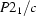

*a* = 15.740 (3) Å
*b* = 10.573 (2) Å
*c* = 10.322 (2) Å
β = 103.86 (3)°
*V* = 1667.8 (6) Å^3^
*Z* = 4
Mo *K*α radiation
μ = 0.21 mm^−1^
*T* = 298 K
0.35 × 0.25 × 0.2 mm

#### Data collection

Stoe IPDS 2T diffractometer
Absorption correction: numerical (*X-SHAPE*; Stoe & Cie, 2005 ▶) *T*_min_ = 0.935, *T*_max_ = 0.957
13245 measured reflections
4474 independent reflections
2439 reflections with *I* > 2σ(*I*)
*R*_int_ = 0.080

#### Refinement

*R*[*F* ^2^ > 2σ(*F* ^2^)] = 0.053
*wR*(*F* ^2^) = 0.143
*S* = 0.92
4474 reflections
219 parameters
H-atom parameters constrained
Δρ_max_ = 0.35 e Å^−3^
Δρ_min_ = −0.28 e Å^−3^

### 

Data collection: *X-AREA* (Stoe & Cie, 2005 ▶); cell refinement: *X-AREA*; data reduction: *X-AREA*; program(s) used to solve structure: *SHELXS97* (Sheldrick, 2008 ▶); program(s) used to refine structure: *SHELXL97* (Sheldrick, 2008 ▶); molecular graphics: *ORTEP-3 for Windows* (Farrugia, 1997 ▶); software used to prepare material for publication: *WinGX* (Farrugia, 1999 ▶).

## Supplementary Material

Crystal structure: contains datablocks I, global. DOI: 10.1107/S1600536811006088/vm2077sup1.cif

Structure factors: contains datablocks I. DOI: 10.1107/S1600536811006088/vm2077Isup2.hkl

Additional supplementary materials:  crystallographic information; 3D view; checkCIF report

## Figures and Tables

**Table 1:** Hydrogen-bond geometry (Å, °) *Cg*1 and *Cg*2 are the centroids of the C1–C3/C5–C7 and C9–C13/C18 rings, respectively.

*D*—H⋯*A*	*D*—H	H⋯*A*	*D*⋯*A*	*D*—H⋯*A*
O3—H3⋯N2	0.82	1.85	2.563 (2)	145
N1—H1*A*⋯O2^i^	0.86	2.36	2.998 (2)	132
C15—H15⋯*Cg*1^ii^	0.92	2.72	3.625 (3)	164
C16—H16⋯*Cg*2^i^	0.92	2.75	3.501 (3)	139

Symmetry codes: (i) 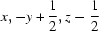; (ii) 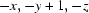.

**Fig. 1.**
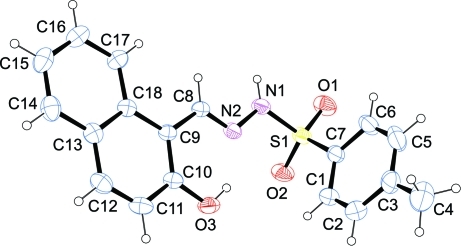
The molecular structure of the title compound, with atom labels and anisotropic displacement ellipsoids (drawn at 30% probability level) for non-H atoms.

**Fig. 2.**
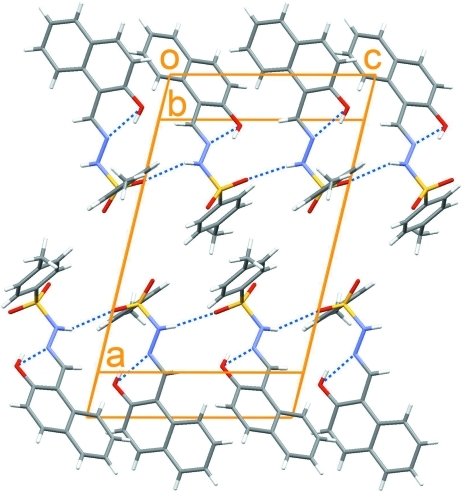
The packing diagram of the title compound. Hydrogen bonds are shown as blue dashed line.

**Fig. 3.**
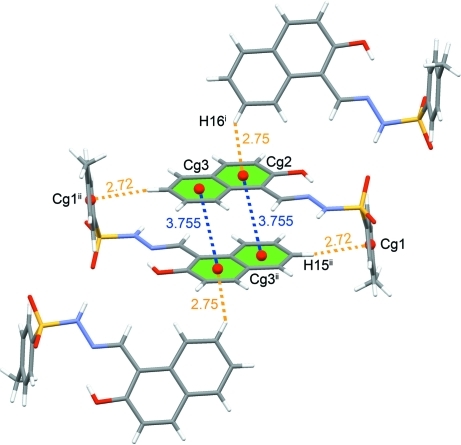
The packing diagram of the title compound showing π-π and C—H···π interactions as blue and orange dashed lines, respectively. Cg1, Cg2 and Cg3 are the centroids of rings C1-C3/C5-C7, C9-C13/C18 and C13-C18, respectively; symmetry codes: (i) x, -y + 1/2, z - 1/2; (ii) -x, -y + 1, -z.).

## supplementary crystallographic information

### Comment

Sulfonyl hydrazones are found to exhibit large medicinal applications. Similar to sulfonamides, sulfonyl hydrazones also have various biological activities. For example, imidosulfonylhydrazones have antibacterial and antineociceptive properties (Silva *et al.*, 2006). Acidic sulfonyl hydrazone derivatives have analgesic and anti-inflammatory activities. 4-Substituted benzenesulfonylhydrazone has been found to have antibacterial activity (Zimmer *et al.*, 1959).

As part of our studies on the synthesis and characterization of hydrazone derivatives (Bikas *et al.*, 2010), we report here the crystal structure of (*E*)-*N*'-((2-hydroxynaphthalen-1-yl)methylene)-4-methylbenzenesulfonohydrazide. The asymmetric unit of the title compound contains one molecule, which is shown in Fig. 1. The packing diagram of the title compound is shown in Fig. 2. The structure is stabilized by an intramolecular O—H···N hydrogen bond, with the nitrogen of the azomethine group (–C=N–) acting as hydrogen bond acceptor and intermolecular N—H···O hydrogen bonds with the S=O group as hydrogen bond acceptor (Table 1 and Fig. 2). The packing is characterized by a π-π interaction between the naphthyl rings (Fig. 3) with *Cg*2···*Cg*3^ii^ distance = 3.7556 (15) Å where *Cg*2 and *Cg*3 are the centroids of C9-C13/C18 and C13-C18, respectively (symmetry code ii: -*x*,1 - *y*,-*z*). Furthermore, C—H_naphthyl_···π_tolyl_ and C—H_naphthyl_···π_naphthyl_ interactions are observed with distances equal to 2.72 and 2.75 Å, respectively (Table 1 & Fig. 3).

### Experimental

All reagents were commercially available and used as received. A methanol (10 ml) solution of 2-hydroxy-1-naphtaldehyde (1.63 mmol) was dropwise added to a methanol solution (10 ml) of 4-methyl-benzenesulfonic acid hydrazide (1.63 mmol), and the mixture was refluxed for 3 hrs. Then the solution was evaporated on a steam bath to 5 ml and cooled to room temperature. A yellow precipitate of the title compound was separated and filtered off, washed with 5 ml of cooled methanol and then dried in air. X-ray quality crystals of the title compound were obtained from methanol by slow solvent evaporation. Yield: 81%, mp: 167.8–168.2 °C.

### Refinement

The hydrogen atom of N—H and O—H group were positioned geometrically and refined as riding atoms with, N—H = 0.86 Å and *U*iso(H) = 1.2 *U*eq(N) and O—H = 0.82 Å and *U*iso(H) = 1.5 *U*eq(O). The C—H protons were positioned geometrically and refined as riding atoms with C—H = 0.93 Å and *U*iso(H) = 1.2 *U*eq(C) for imine and aromatic C—H groups and C—H = 0.96 Å and *U*iso(H) = 1.5 *U*eq(C) for methyl group.

### Crystal data

**Table:**

C_18_H_16_N_2_O_3_S	*F*(000) = 712
*M**_r_* = 340.40	*D*_x_ = 1.356 Mg m^−^^3^
Monoclinic, *P*2_1_/*c*	Mo *K*α radiation, λ = 0.71073 Å
Hall symbol: -P 2ybc	Cell parameters from 4474 reflections
*a* = 15.740 (3) Å	θ = 2.3–29.2°
*b* = 10.573 (2) Å	µ = 0.21 mm^−^^1^
*c* = 10.322 (2) Å	*T* = 298 K
β = 103.86 (3)°	Plate, yellow
*V* = 1667.8 (6) Å^3^	0.35 × 0.25 × 0.2 mm
*Z* = 4	

### Data collection

**Table:**

Stoe IPDS 2T diffractometer	4474 independent reflections
Radiation source: fine-focus sealed tube	2439 reflections with *I* > 2σ(*I*)
graphite	*R*_int_ = 0.080
Detector resolution: 0.15 mm pixels mm^-1^	θ_max_ = 29.2°, θ_min_ = 2.3°
rotation method scans	*h* = −21→21
Absorption correction: numerical (*X-SHAPE*; Stoe & Cie, 2005)	*k* = −14→13
*T*_min_ = 0.935, *T*_max_ = 0.957	*l* = −13→14
13245 measured reflections	

### Refinement

**Table:**

Refinement on *F*^2^	Primary atom site location: structure-invariant direct methods
Least-squares matrix: full	Secondary atom site location: difference Fourier map
*R*[*F*^2^ > 2σ(*F*^2^)] = 0.053	Hydrogen site location: inferred from neighbouring sites
*wR*(*F*^2^) = 0.143	H-atom parameters constrained
*S* = 0.92	*w* = 1/[σ^2^(*F*_o_^2^) + (0.0771*P*)^2^] where *P* = (*F*_o_^2^ + 2*F*_c_^2^)/3
4474 reflections	(Δ/σ)_max_ < 0.001
219 parameters	Δρ_max_ = 0.35 e Å^−^^3^
0 restraints	Δρ_min_ = −0.28 e Å^−^^3^

### Special details

**Table:**

Geometry. All e.s.d.'s (except the e.s.d. in the dihedral angle between two l.s. planes) are estimated using the full covariance matrix. The cell e.s.d.'s are taken into account individually in the estimation of e.s.d.'s in distances, angles and torsion angles; correlations between e.s.d.'s in cell parameters are only used when they are defined by crystal symmetry. An approximate (isotropic) treatment of cell e.s.d.'s is used for estimating e.s.d.'s involving l.s. planes.
Refinement. Refinement of *F*^2^ against ALL reflections. The weighted *R*-factor *wR* and goodness of fit *S* are based on *F*^2^, conventional *R*-factors *R* are based on *F*, with *F* set to zero for negative *F*^2^. The threshold expression of *F*^2^ > σ(*F*^2^) is used only for calculating *R*-factors(gt) *etc*. and is not relevant to the choice of reflections for refinement. *R*-factors based on *F*^2^ are statistically about twice as large as those based on *F*, and *R*- factors based on ALL data will be even larger.

### Fractional atomic coordinates and isotropic or equivalent isotropic displacement parameters (Å^2^)

**Table:**

	*x*	*y*	*z*	*U*_iso_*/*U*_eq_	
C6	0.41858 (14)	0.4904 (3)	0.3196 (2)	0.0705 (8)	
H6	0.4426	0.4414	0.2626	0.085*	
S1	0.34568 (3)	0.27206 (7)	0.38051 (5)	0.0577 (2)	
O2	0.31790 (11)	0.23002 (19)	0.49498 (15)	0.0713 (5)	
N1	0.26101 (10)	0.25594 (19)	0.25177 (17)	0.0534 (5)	
H1A	0.2632	0.2147	0.1808	0.064*	
O1	0.41463 (10)	0.2084 (2)	0.34045 (19)	0.0783 (6)	
N2	0.18558 (10)	0.31574 (17)	0.26935 (16)	0.0474 (4)	
C8	0.11925 (12)	0.3191 (2)	0.16968 (19)	0.0436 (5)	
H8	0.1228	0.2816	0.0896	0.052*	
C9	0.03862 (12)	0.38038 (19)	0.17943 (18)	0.0414 (4)	
O3	0.10297 (10)	0.45363 (19)	0.40269 (14)	0.0656 (5)	
H3	0.1464	0.4217	0.3853	0.098*	
C18	−0.03826 (12)	0.37537 (19)	0.07041 (19)	0.0429 (4)	
C7	0.36860 (12)	0.4348 (3)	0.39778 (19)	0.0553 (6)	
C17	−0.04179 (15)	0.3112 (2)	−0.0504 (2)	0.0539 (6)	
H17	0.0077	0.2688	−0.0618	0.065*	
C10	0.03447 (13)	0.4460 (2)	0.29415 (19)	0.0489 (5)	
C1	0.33366 (15)	0.5089 (3)	0.4823 (2)	0.0660 (7)	
H1	0.3007	0.4723	0.5361	0.079*	
C11	−0.04155 (15)	0.5093 (2)	0.3048 (2)	0.0610 (6)	
H11	−0.0425	0.5538	0.3821	0.073*	
C15	−0.19112 (16)	0.3721 (3)	−0.1373 (3)	0.0675 (7)	
H15	−0.2415	0.3704	−0.2062	0.081*	
C13	−0.11516 (13)	0.4395 (2)	0.0837 (2)	0.0500 (5)	
C14	−0.19068 (14)	0.4359 (3)	−0.0229 (3)	0.0637 (7)	
H14	−0.2410	0.4779	−0.0145	0.076*	
C12	−0.11397 (15)	0.5057 (2)	0.2025 (2)	0.0598 (6)	
H12	−0.1641	0.5481	0.2110	0.072*	
C16	−0.11621 (17)	0.3097 (3)	−0.1513 (2)	0.0653 (7)	
H16	−0.1166	0.2664	−0.2299	0.078*	
C3	0.39614 (15)	0.6954 (3)	0.4084 (3)	0.0700 (8)	
C5	0.43222 (16)	0.6196 (3)	0.3274 (3)	0.0787 (9)	
H5	0.4668	0.6565	0.2762	0.094*	
C2	0.34799 (17)	0.6369 (3)	0.4863 (3)	0.0738 (8)	
H2	0.3243	0.6860	0.5437	0.089*	
C4	0.4090 (2)	0.8355 (4)	0.4087 (4)	0.0969 (10)	
H4A	0.3725	0.8711	0.3290	0.145*	
H4B	0.4692	0.8539	0.4117	0.145*	
H4C	0.3937	0.8715	0.4854	0.145*	

### Atomic displacement parameters (Å^2^)

**Table:**

	*U*^11^	*U*^22^	*U*^33^	*U*^12^	*U*^13^	*U*^23^
C6	0.0495 (12)	0.105 (3)	0.0628 (15)	0.0036 (13)	0.0256 (11)	0.0034 (14)
S1	0.0488 (3)	0.0843 (5)	0.0418 (3)	0.0121 (3)	0.0145 (2)	0.0078 (3)
O2	0.0796 (10)	0.0930 (15)	0.0447 (9)	0.0094 (9)	0.0215 (8)	0.0149 (8)
N1	0.0489 (9)	0.0698 (14)	0.0440 (9)	0.0081 (8)	0.0159 (7)	−0.0049 (9)
O1	0.0576 (9)	0.1076 (16)	0.0717 (11)	0.0291 (9)	0.0196 (8)	0.0054 (10)
N2	0.0468 (8)	0.0557 (12)	0.0426 (9)	0.0048 (7)	0.0168 (7)	0.0009 (8)
C8	0.0503 (10)	0.0455 (13)	0.0370 (10)	0.0002 (9)	0.0145 (8)	0.0005 (8)
C9	0.0483 (10)	0.0396 (12)	0.0377 (9)	0.0012 (8)	0.0133 (8)	0.0014 (8)
O3	0.0641 (9)	0.0914 (14)	0.0413 (8)	0.0074 (9)	0.0124 (7)	−0.0137 (8)
C18	0.0508 (10)	0.0363 (12)	0.0429 (10)	0.0012 (8)	0.0138 (8)	0.0052 (8)
C7	0.0388 (9)	0.0851 (19)	0.0410 (10)	0.0006 (10)	0.0075 (8)	0.0010 (11)
C17	0.0605 (12)	0.0487 (14)	0.0489 (12)	0.0027 (10)	0.0059 (9)	−0.0041 (10)
C10	0.0554 (11)	0.0537 (14)	0.0398 (10)	0.0019 (9)	0.0157 (9)	0.0001 (9)
C1	0.0621 (13)	0.092 (2)	0.0478 (12)	−0.0105 (13)	0.0207 (10)	−0.0082 (12)
C11	0.0748 (15)	0.0609 (17)	0.0533 (13)	0.0125 (12)	0.0269 (11)	−0.0063 (11)
C15	0.0601 (13)	0.0675 (19)	0.0645 (15)	−0.0075 (12)	−0.0053 (11)	0.0088 (13)
C13	0.0517 (11)	0.0447 (14)	0.0549 (12)	0.0014 (9)	0.0151 (9)	0.0091 (10)
C14	0.0495 (11)	0.0630 (18)	0.0763 (16)	0.0046 (11)	0.0106 (11)	0.0169 (13)
C12	0.0584 (12)	0.0594 (16)	0.0669 (15)	0.0153 (11)	0.0255 (11)	0.0050 (12)
C16	0.0759 (15)	0.0604 (17)	0.0513 (13)	−0.0027 (12)	−0.0011 (11)	−0.0032 (11)
C3	0.0472 (12)	0.096 (2)	0.0622 (15)	−0.0087 (12)	0.0032 (11)	0.0008 (14)
C5	0.0523 (13)	0.105 (3)	0.0819 (19)	−0.0119 (14)	0.0222 (13)	0.0181 (17)
C2	0.0712 (15)	0.094 (2)	0.0565 (14)	−0.0097 (15)	0.0160 (12)	−0.0129 (14)
C4	0.0861 (19)	0.099 (3)	0.098 (2)	−0.0163 (18)	0.0068 (17)	0.0056 (19)

### Geometric parameters (Å, °)

**Table:**

C6—C5	1.383 (4)	C10—C11	1.398 (3)
C6—C7	1.386 (3)	C1—C2	1.370 (4)
C6—H6	0.9300	C1—H1	0.9300
S1—O1	1.4204 (17)	C11—C12	1.356 (3)
S1—O2	1.4257 (17)	C11—H11	0.9300
S1—N1	1.6499 (18)	C15—C14	1.359 (4)
S1—C7	1.758 (3)	C15—C16	1.389 (4)
N1—N2	1.395 (2)	C15—H15	0.9300
N1—H1A	0.8600	C13—C12	1.408 (3)
N2—C8	1.279 (2)	C13—C14	1.413 (3)
C8—C9	1.450 (3)	C14—H14	0.9300
C8—H8	0.9300	C12—H12	0.9300
C9—C10	1.387 (3)	C16—H16	0.9300
C9—C18	1.443 (3)	C3—C5	1.375 (4)
O3—C10	1.358 (2)	C3—C2	1.376 (4)
O3—H3	0.8200	C3—C4	1.496 (5)
C18—C17	1.409 (3)	C5—H5	0.9300
C18—C13	1.422 (3)	C2—H2	0.9300
C7—C1	1.382 (3)	C4—H4A	0.9600
C17—C16	1.368 (3)	C4—H4B	0.9600
C17—H17	0.9300	C4—H4C	0.9600
			
C5—C6—C7	119.2 (3)	C7—C1—H1	120.3
C5—C6—H6	120.4	C12—C11—C10	120.1 (2)
C7—C6—H6	120.4	C12—C11—H11	120.0
O1—S1—O2	120.06 (12)	C10—C11—H11	120.0
O1—S1—N1	104.04 (10)	C14—C15—C16	119.9 (2)
O2—S1—N1	106.64 (10)	C14—C15—H15	120.1
O1—S1—C7	109.94 (12)	C16—C15—H15	120.1
O2—S1—C7	108.50 (11)	C12—C13—C14	121.6 (2)
N1—S1—C7	106.82 (10)	C12—C13—C18	119.13 (18)
N2—N1—S1	113.38 (13)	C14—C13—C18	119.3 (2)
N2—N1—H1A	123.3	C15—C14—C13	121.2 (2)
S1—N1—H1A	123.3	C15—C14—H14	119.4
C8—N2—N1	117.67 (17)	C13—C14—H14	119.4
N2—C8—C9	121.08 (18)	C11—C12—C13	121.7 (2)
N2—C8—H8	119.5	C11—C12—H12	119.2
C9—C8—H8	119.5	C13—C12—H12	119.2
C10—C9—C18	118.81 (18)	C17—C16—C15	120.6 (2)
C10—C9—C8	120.25 (17)	C17—C16—H16	119.7
C18—C9—C8	120.94 (17)	C15—C16—H16	119.7
C10—O3—H3	109.5	C5—C3—C2	117.3 (3)
C17—C18—C13	117.44 (18)	C5—C3—C4	120.1 (3)
C17—C18—C9	123.77 (18)	C2—C3—C4	122.5 (3)
C13—C18—C9	118.79 (18)	C3—C5—C6	122.0 (3)
C1—C7—C6	119.7 (3)	C3—C5—H5	119.0
C1—C7—S1	121.08 (19)	C6—C5—H5	119.0
C6—C7—S1	119.2 (2)	C1—C2—C3	122.4 (3)
C16—C17—C18	121.6 (2)	C1—C2—H2	118.8
C16—C17—H17	119.2	C3—C2—H2	118.8
C18—C17—H17	119.2	C3—C4—H4A	109.5
O3—C10—C9	122.90 (18)	C3—C4—H4B	109.5
O3—C10—C11	115.60 (19)	H4A—C4—H4B	109.5
C9—C10—C11	121.50 (19)	C3—C4—H4C	109.5
C2—C1—C7	119.4 (2)	H4A—C4—H4C	109.5
C2—C1—H1	120.3	H4B—C4—H4C	109.5
			
O1—S1—N1—N2	−178.38 (16)	C8—C9—C10—C11	178.0 (2)
O2—S1—N1—N2	53.77 (18)	C6—C7—C1—C2	0.9 (3)
C7—S1—N1—N2	−62.10 (17)	S1—C7—C1—C2	−175.48 (18)
S1—N1—N2—C8	173.14 (15)	O3—C10—C11—C12	−179.0 (2)
N1—N2—C8—C9	−179.48 (18)	C9—C10—C11—C12	1.1 (4)
N2—C8—C9—C10	5.5 (3)	C17—C18—C13—C12	−179.4 (2)
N2—C8—C9—C18	−175.04 (19)	C9—C18—C13—C12	0.0 (3)
C10—C9—C18—C17	−179.7 (2)	C17—C18—C13—C14	0.2 (3)
C8—C9—C18—C17	0.8 (3)	C9—C18—C13—C14	179.6 (2)
C10—C9—C18—C13	0.9 (3)	C16—C15—C14—C13	−0.4 (4)
C8—C9—C18—C13	−178.56 (19)	C12—C13—C14—C15	179.7 (2)
C5—C6—C7—C1	−0.3 (3)	C18—C13—C14—C15	0.1 (4)
C5—C6—C7—S1	176.16 (18)	C10—C11—C12—C13	−0.1 (4)
O1—S1—C7—C1	−154.14 (17)	C14—C13—C12—C11	180.0 (2)
O2—S1—C7—C1	−21.0 (2)	C18—C13—C12—C11	−0.4 (4)
N1—S1—C7—C1	93.57 (18)	C18—C17—C16—C15	0.1 (4)
O1—S1—C7—C6	29.5 (2)	C14—C15—C16—C17	0.3 (4)
O2—S1—C7—C6	162.58 (17)	C2—C3—C5—C6	2.3 (4)
N1—S1—C7—C6	−82.81 (18)	C4—C3—C5—C6	−177.0 (2)
C13—C18—C17—C16	−0.3 (3)	C7—C6—C5—C3	−1.4 (4)
C9—C18—C17—C16	−179.7 (2)	C7—C1—C2—C3	0.1 (4)
C18—C9—C10—O3	178.6 (2)	C5—C3—C2—C1	−1.7 (4)
C8—C9—C10—O3	−1.9 (3)	C4—C3—C2—C1	177.6 (2)
C18—C9—C10—C11	−1.5 (3)		

### Hydrogen-bond geometry (Å, °)

**Table:**

Cg1 and Cg2 are the centroids of the C1–C3/C5–C7 and C9–C13/C18 rings, respectively.

**Table:**

*D*—H···*A*	*D*—H	H···*A*	*D*···*A*	*D*—H···*A*
O3—H3···N2	0.82	1.85	2.563 (2)	145.
N1—H1A···O2^i^	0.86	2.36	2.998 (2)	132.
C15—H15···Cg1^ii^	0.92	2.72	3.625 (3)	164
C16—H16···Cg2^i^	0.92	2.75	3.501 (3)	139

Symmetry codes: (i) *x*, −*y*+1/2, *z*−1/2; (ii) −*x*, −*y*+1, −*z*.
